# Effectiveness of a parental training programme in enhancing the parent–child relationship and reducing harsh parenting practices and parental stress in preparing children for their transition to primary school: a randomised controlled trial

**DOI:** 10.1186/1471-2458-13-1079

**Published:** 2013-11-16

**Authors:** Ho Cheung William Li, Sophia SC Chan, Yim Wah Mak, Tai Hing Lam

**Affiliations:** 1 School of Nursing, The University of Hong Kong, 4/F, William M. W. Mong Block, 21 Sassoon Road, Pokfulam, Hong Kong; 2 School of Nursing, The Hong Kong Polytechnic University, Hung Hom, Hong Kong; 3 School of Public Health, The University of Hong Kong, Pokfulam, Hong Kong

**Keywords:** Children, Harsh parenting practices, Parent–child relationships, Parental stress, Transition

## Abstract

**Background:**

Entering primary school is an important childhood milestone, marking the beginning of a child’s formal education. Yet the change creates a time of vulnerability for the child, the parents and the parent–child relationship. Failure to adjust to the transition may place the family in a psychologically devastating position. The aims of this study were to test the effectiveness of a parental training programme in enhancing the parent–child relationship and decreasing parental stress by reducing harsh parenting in preparing children for the transition to primary school.

**Methods:**

A randomised controlled trial incorporating a two-group pre-test and repeated post-test was conducted in one of the largest public housing estates in Hong Kong. A total of 142 parents were recruited, with 72 parents randomly assigned to the experimental group and 70 to the control group. Harsh parenting practices, parent–child relationships and parental stress were assessed.

**Results:**

In comparison to parents in the control group, those in the experimental group engaged in less harsh parenting practices and reported better parent–child relationships. However, parental stress scores did not differ significantly between the two groups.

**Conclusion:**

This study addressed a gap in the literature by examining the effectiveness of the training programme for enhancing parent–child relationship and decreasing parental stress at the time of a child’s transition to primary school. The findings from this study provide empirical evidence of the effectiveness of the parental training programme and highlight the significance of parenting in promoting a smooth transition for children from kindergarten to primary 1.

**Trial registration:**

ClinicalTrials.gov: NCT01845948.

## Background

Moving from kindergarten to primary 1 is perceived as one of the most important milestones for children, marking the beginning of their formal education. This transition can be both an exciting and an anxiety-provoking experience for children, having a profound effect on them [1-3]. A recent study showed that the transition creates stress for children, as evidenced by high levels of worry and low levels of happiness before the start of the academic year in primary school [4]. Furthermore, the transition may not only have an effect on the children but also create stress within the families because parents need to adjust themselves to new roles. In contemporary Chinese society in particular, most men and women must work full time outside their homes and take up multiple social roles as paid workers, spouses and parents. Tang [5] reported that due to insufficient skills, time and energy to fulfil such demanding roles, many Chinese parents experience considerable stress. When children move from kindergarten to primary school, the parents’ concerns shift — from their child’s physical well-being to their child’s future success. In this circumstance, some parents may adopt components of a harsh parenting style in rearing their children, which may actually cause some harm to the family. According to Shek [6], harshness in fathers and a demanding style in mothers can trigger child–parent conflicts, with the relationship being bidirectional. Furthermore, parental criticism not only increases conflicts in the family but can also incite emotional and aggressive behaviour in the child [7]. A previous study showed that conflicts within the family were positively related to children’s emotional problems, whereas positive parent–child relationships were inversely related to such problems [8]. Some parents may believe, however, that offering praise when their children do well will cause the children to fail because they will become overly proud of themselves. However, lack of praise may decrease parental warmth, which is an essential element in promoting emotional adjustment and social and scholastic achievement in children [9].

Hong Kong has witnessed a significant change in family structure, with extended families gradually being replaced by small nuclear families over the past few decades [10]. Children are thus receiving more attention from parents as a result of there being fewer, if any, siblings in the nuclear family. In addition, most parents and school children in Hong Kong share a belief that a desirable career and bright future are the inevitable results of strong academic achievement. There is a famous Chinese proverb that says, ‘In textbooks you will find girls with complexions like jade and houses made of gold’. Under the influence of such traditional Chinese thought, children in Hong Kong are under sustained and substantial pressure within the family and at school while growing up [11]. Meanwhile, Hong Kong Chinese parents are also exposed to considerable stress when their child is promoted from kindergarten to primary 1 education.

The transition creates a time of vulnerability for the child, the parents and the child–parent relationship. Failure to adjust to the transition, on the part of either the parents or the child, might place the family in a psychologically devastating position. There is some evidence that good provision for transitions will result in fewer difficulties encountered by children in later school life [3]. The literature highlights that parental involvement in the preparation of children plays a significant role in ensuring a successful transition [12]. Indeed, if the transition can be handled in an appropriate manner, it can be regarded as a valuable opportunity for children to learn not only how to solve problems, but also how to develop confidence in response to a changing environment. Such experiences will lay a good foundation for their future learning and promote their interest in life-long learning. Therefore, before the admission to primary school, providing interventions that can help children and parents adjust to their new roles and developmental tasks during the stressful school transition period is crucial.

Studies conducted in Western countries have shown that programmes focusing on parenting are beneficial to the psychological well-being of both children and parents [3,13-16]. One such programme is the Triple P-Positive Parenting Programme (the Triple P system, TPS), which aims to bring about positive psychological outcomes by promoting positive parenting. Sanders et al. [3] investigated the effectiveness of the TPS in reducing the prevalence of children’s mental health problems, parental adjustment difficulties and dysfunctional parenting at the time of transition. They found that this programme was able to reduce the emotional problems and psychosocial difficulties for children living in TPS communities. In addition, parents who followed the programme were less likely to engage in coercive parenting or experience stress and/or depressive episodes. Consistent findings were obtained by Beckett et al. [14], who examined the effectiveness of different types of parenting programmes to improve the social behaviour and reading ability of children at risk. The results indicated that compared with parents in the control group, parents who followed a programme that focused on building a better parent–child relationship (Incredible Years) tended to report their children as having fewer behavioural problems. In addition, these parents felt more able to manage their child’s behavioural problems. Thus, there is compelling evidence to support the importance of parenting in reducing the psychological and emotional problems of both young children and their parents, potentially helping them to go through a school transition.

The most common primary 1 preparatory programme run by schools in Hong Kong teaches children how to organise their school bags, observe school rules and regulations, familiarise themselves with school timetables and different subjects and record homework in their student handbooks [17]. Thus, the programme activities focus on the physical preparation of children, with the importance of parental involvement and the psycho-educational needs of parents during the process of transition often overlooked by schools and by the parents themselves. Indeed, there is a need for parental training programmes on the preparation of children for transition to primary schools in Hong Kong.

Although the programmes implemented in Western countries show promising results in promoting the psychological well-being of children and parents at the time of transition, these programmes may not be applicable to parents in Chinese society because of the different cultural context. As a former British colony, Hong Kong people have been subject to the effect of Westernisation. However, many of them are still rooted in Chinese culture because of the influence of Confucianism [18], which emphasises governance and obedience [19]. Therefore, parents in Hong Kong commonly believe in the notion of ‘spare the rod and spoil the child', making them unable to recognise the harmful effects of harsh parenting [20]. In addition, some of the gestures (like hugging and kissing) that show parental warmth are unlikely to be adopted by parents in Chinese society because these parents are considerably more reserved than parents in Western countries [21]. Hence, rather than promoting positive parenting, discouraging harsh parenting seems to be a more feasible and suitable approach for Chinese parents.

Given the above issues, there is a great need to develop and evaluate appropriate interventions for Chinese parents so that they can help their children enjoy a smooth passage to a pleasurable learning life in primary school, which will come about by building good parent–child relationships and decreasing parental stress. The aims of this study were to test the effectiveness of a parental training programme in improving the parent–child relationship and decreasing parental stress by reducing harsh parenting at the time of transition.

### Hypotheses of the intervention

The three hypotheses were:

1: Parents who received the parental training programme would engage in less harsh parenting practices when compared with parents who received assessment only.

2: Parents who received the parental training programme would report better parent–child relationships when compared with parents who received assessment only.

3: Parents who received the parental training programme would reduce the level of stress during the transition when compared with parents who received assessment only.

#### Theoretical framework

The importance of the parent–child relationship during transition can be accounted for within Lazarus and Folkman’s [22] framework of cognitive appraisal, stress and coping. According to this framework, the emotional experience of an individual towards a potentially stressful event is determined by that individual’s primary and secondary appraisals. Primary appraisal refers to the individual’s evaluation of the meaning and significance of the event as it pertains to the individual’s well-being. Those events that are perceived by the individual as being important and relevant are likely to provoke emotional stress. Secondary appraisal refers to the individual’s assessment of his or her capability to cope with such an event. Those individuals with more available resources are likely to have better control over stressful situations, resulting in minimal psychological distress. Indeed, social support from parents has long been recognised as an important resource in secondary appraisal [23,24]. Specifically, parents can promote effective coping in their children via coaching (listening to children’s voices and providing feedback) and modelling (setting examples of coping behaviour). These strategies can enable children to consider every possible solution to deal with stressful situations and can lessen the effect of such situations on the children’s psychological health [23]. Therefore, it is anticipated that children who have a positive relationship with their parents are most likely to receive social support from them, resulting in a smooth transition to primary school life.

Most social cognitive theories assume that intention to change is the best predictor of actual change. However, individuals often do not behave in accordance with their intentions. Examples include habitual smokers and persons who are obese. There is increasing evidence that the Health Action Process Approach (HAPA) is helpful in explaining the psychological mechanisms in bridging the gap between intention and actual change in health behaviour. This study was guided by the HAPA, which has been widely used to address the gap between intention to change and a person’s actual change in behaviour [25-27]. The HAPA model was originally adopted in changing health-compromising behaviour by using an inductive approach involving two phases: the pre-intentional motivation process and the volition process. The former process emphasises raising an individual’s intention either to engage in a preventive measure or to modify risky behaviour in favour of a healthy practice. The volition process is self-regulatory and determined by action plans and action control. These two mediators help the individual to structure a strategy for maintaining healthy practices and defeating other conflicting ideas [28,29]. Indeed, harsh parenting can be regarded as health-compromising behaviour within a family. It is well documented that most parents comprehend the importance of positive parenting, but regrettably find it difficult to refrain from harsh parenting [30]. This has important implications for restructuring a new parenting programme. Through incorporating a HAPA to such a programme, it is expected that parents will engage in positive parenting and develop strategies to minimise negative parenting by motivating them to put positive parenting into practice.

## Methods

### Design

This study was carried out in one of the largest public housing estates in Hong Kong. A randomized controlled trial, two-group pre-test and repeated post-test, between-subjects design was employed. A simple complete randomization was adopted in this study. We put two sealed opaque envelopes in a box, with one labelled experimental and another labelled control. Each parent was asked to draw one envelop from the box to determine group assignment. Another sealed opaque envelop with the same group assignment being drawn was then put back in the box again before the next assignment. A single-blinded technique was used, in which the person who was responsible for data collecting, was unaware of the intervention allocation of the study participants.

### Participants

Chinese parents living in Tung Chung with a child to be promoted from kindergarten to primary one education, and meeting the inclusion criteria for the study, were invited to participate in it. The criteria were the following: (1) parent must be able to read and write Chinese, and (2) parent must have had primary school education or above. Parents with identified cognitive and learning problems were excluded, as were children with such problems. A convenience sampling of 142 parents was recruited during the school summer holidays in 2009.

### Intervention

In the control group, parents had no intervention except that an information leaflet for parents on helping children to adapt to the new primary school life published by Education Bureau was given to each parent at the end of data collection. The content of the information leaflet include the following:

•the aims of primary education;

•the layout of the school and its facilities;

•the details of school life, including timetabling, school regulations, homework policies, use of textbooks/learning packages and exercise books, assessment methods, policies and procedures on sick leave, and school routines;

•the mode of home−school collaboration.

In the experimental group, parents participated in the parental training programme approximately 1 month before the start of the academic year in primary school. Two social workers, each with a minimum of 5 years’ experience in providing family counselling, implemented the interventions. The parental training programme was set up for small groups of 8 to 12 parents each and ran for 4 consecutive weeks. The programme consisted of four group sessions, each lasting about 2 hours. Overall, the parental intervention included teaching parents (1) to use more active listening skills, (2) to engage less in harsh parenting practices, (3) to use more praise and encouragement and (4) to set reasonable expectations in the rearing of their children. Each session began with a review of the skills or concepts discussed in the previous sessions, i.e., each session built upon the previous session. The following three approaches were used to facilitate the learning process:

•Metaphor: by using the living plant as a symbol of growth and nurturing, the concept of parenting becomes easy for parents to master.

•Teaching without a teacher: this means encouraging parents to learn from each other through group discussion.

•Role playing and planning: parents have the opportunity to act out roles in front of group members. They can then refine their parenting plan and skills and gain the self-confidence to actualise the plan with their children.

The parental training programme, which aims to enhance the parent–child relationship and reduce parental stress at the time of transition, was developed by the research team. The intervention components were selected on the basis of the theoretical framework of parenting styles put forth by Baumrind [31]. Under this framework, harsh parenting is a child rearing style that communicates high levels of demandingness but low levels of responsiveness. It is characterised by the use of corporal punishment or verbal hostility to convey high expectations without any explanation [31,32]. In fact, according to the results of a published study, active listening and praising are positively associated with responsiveness, whereas setting reasonable expectations is negatively correlated with demandingness [32]. Therefore, the research team decided to include these components in the intervention to reduce harsh parenting.

The development of the intervention was based on the HAPA model, according to which risk perception is a distal antecedent for raising the intention to change in the pre-intentional motivation process. In the initial motivation stage, a parent might have already developed an intention to help his/her child adapt the transition. Within this stage, risk awareness is seen as a distal antecedent (for example, ‘My child and I are at risk of family health problems or disturbances in the family harmony or family happiness if the transition does not go well’). Parents in the group were therefore invited to discuss any potential problems that the family or child might encounter during the transition. This helped the parents to become aware of the negative consequences of a maladaptive transition and subsequently develop an intention to help their children through the transition. Further elaboration of the parents’ thoughts about the consequences and positive outcome expectancies in smoothing the transition was then facilitated.

In applying the HAPA to the parenting programme, self-regulation was emphasised in the motivation process, whereby role playing, demonstration and re-demonstration were applied. Parents were invited to demonstrate a quarrel they might have with their child and the usual ways they would deal with it. Other parents in the group were invited to suggest some alternative ways for handling the quarrel and techniques the role-players could use to control their own emotions. These activities helped parents to structure an appropriate strategy to refrain from harsh parenting practices and maintain a positive attitude.

The programme was designed as a cognitive-dissonance-based intervention for use in group therapy to help parents build supportive relationships with their children and thus help the families smoothen the transition to primary 1 education. Parents were encouraged to enhance their knowledge and parenting skills through an inductive learning approach. Parent–child relationships could then be enhanced by changing the attending parents’ parenting practices. Several goals may be particularly important in preventing adjustment problems:

•Increasing parents’ awareness of what the children need in the transition.

•Motivating parents to change to and maintain positive parenting by increasing their risk awareness and reinforcing the consequences of harsh parenting.

•Enhancing positive parent–child interactions and relationship-building between the parents and child via warm parenting, positive parent–child interactions and use of praise.

•Helping parents identify high-risk situations during the transition that might create adjustment difficulties, such as writing, doing homework and performing other school tasks.

•Facilitating development, planning, rehearsing and revising parental strategies to smooth the transition.

The protocol, which covers the schedule of activities and the session contents, is described in Table 1.

**Table 1:** Intervention protocol for parents

**Session**	**Objectives**	**Activities**
One	1. To understand children need from parents when they move to primary school;	• Introduction: Introduce the objectives and general rules of the programme. Parents are encouraged to be active in participation in group activities, such as role play and discussion.
		• Sharing: Facilitators invite parents to share how they interact with their child at home.
		• Role play: Facilitators invite two parents to demonstrate an interaction between parent and child. Scenario: The child reported the difficulties encountered in the school to a parent.
		• Group discussion: Identify strategies to enhance smooth interaction between parent and child.
	2. To learn the importance of parent involvement in preparing, and dealing with the transition;	
		• Round up: Facilitators sum up the content that discussed in this session and encourage parents to practice the skills at home.
	3. To learn the important skills of active listening	
Two	1. To learn the consequences of harsh parenting	• Review: Facilitators review the skills or concepts discussed in previous sessions
		• Sharing: Facilitators invite parents to share their methods in rearing their children
	2. To engage in less harsh parenting practices	
		• Role play: Scenario: Parents were invited to demonstrate a quarrel they might have with their child and the usual ways they dealt with it.
		• Group discussion: Alternative ways for handling the quarrel and controlling emotions.
		• Round up: Facilitators sum up the content that discussed in this session and encourage parents to engage in less harsh parenting practices at home.
Three	1. To learn positive interaction	• Review: Facilitators review the skills or concepts discussed in previous sessions
	2. To learn and practice parenting skill: expressing love and concern; acknowledge efforts taking the point of view of their children and praise their children	• Sharing: Facilitators invite parents to share whether they would often use praise and encouragement in rearing their children.
		• Role play: Interaction between parent and child. Scenario: When receiving unfavourable report about the child’s school performance.
		• Group discussion: The pros and cons of using praise and encouragement in rearing their children.
		• Round up: Facilitators sum up the content that discussed in this session and encourage parents to use more praise and encourage.
Four	To set reasonable expectations of their children	• Review: Facilitators review the skills or concepts discussed in previous sessions.
	To learn positive interaction when dealing with their children’s homework, tests and examination	• Sharing: Facilitators invite parents to share their expectations of their children.
		• Group discussion: What are the consequence of having unreasonable expectations of their children.
		• Exercise: Facilitators ask parents to think about and write down their own reasonable expectations of their children.
		• Round up: Facilitators sum up the content that discussed in this session and encourage parents to set reasonable expectations of their children.

To ensure the integrity of the intervention, the same social workers were responsible for the parental training. These social workers were required to complete a 2-hour training workshop that was provided by the principal investigator. The workshop covered children’s psychological needs, information regarding positive and negative parenting, practical tips and counselling techniques. The social workers were also required to record the total time spent in each parental training session.

Concerning the quality of the intervention, an intervention component checklist was developed by the research team. Ten intervention sessions were randomly chosen to be videotaped. The tapes were then audited by the research team using the checklist. Comments and suggestions were given to the social workers in the regular weekly evaluation meetings.

### Measures

#### Self-reported parent–child relationship

The parent–child relationship of the participants was measured by using a self-reporting method consisting of two items, with one asking “How satisfied are you with the parent–child relationship?” and the other ‘As a parent, how satisfied are you with yourself?’ Each item is rated on a 6-point Likert scale ranging from 1 (total unsatisfactory) to 6 (total satisfactory). A total is calculated by adding the two items together to obtain a score ranging from 2 to 12, with higher scores indicating better parent–child relationships.

#### Chinese version of perceived parental aggression scale

Perceived parental aggression scale is one of the subscales of Parental Acceptance Rejection Questionnaire [33], which is a parent self-report instrument that measures harsh parenting, i.e. physical or verbal aggression towards children. It contains 15 items and each item is rated on a 5-point Likert scale ranging from 1 (never) to 5 (always). A total is calculated by adding all items together to obtain a score ranging from 15 to 75, with higher scores indicating higher levels of hostility and aggression to the child.

The psychometric properties of the perceived parental aggression scale have been empirically tested [33,34], showing adequate reliability and validity.

#### Parental Stress Scale (PSS)

The PSS was developed by Berry and Jones [35] to assess a parent’s subjective feelings of strain, difficulty and dissatisfaction in reaction to stressors in the parent–child relationship. The PSS consists of 18 items on the perception of parental stress, using a 5-point Likert scale from 1 to 5 (1 = strongly disagree, 2 = disagree, 3 = somewhat disagree, 4 = agree, 5 = strongly agree). A total is calculated by adding all items together to obtain a score ranging from 18 to 90, with higher scores indicating higher levels of parental stress.

The psychometric properties of the PSS have been empirically tested [35-37], showing good concurrent validity, excellent construct validity and adequate internal consistency reliability. The Chinese version of the PSS has been translated and used by Cheung [36], the findings demonstrating acceptable psychometric properties for researchers to assess stress among Chinese parents.

### Data collection

Recruitment and data collection were conducted during the summer in 2009. Participants were recruited through referrals from the Hong Kong Sheung Kung Hui Welfare Council in Tung Chung. Prior to the study, ethical approval was obtained from the Institutional Review Board of the University of Hong Kong. The centre’s head of Sheng Kung Hui integrated services in Tung Chung was fully informed of the study’s purpose, nature, design and duration. Written consent was obtained from parents after they were told the purposes of the study and agreed to participate. Parents were told that they were under no obligation to participate, could withdraw from the study with impunity at any time and were assured of the confidentiality of the data to be collected.

After they had signed consent forms, a research assistant collected demographic and baseline data from parents. Data collection was divided into three phases: at the time of recruitment (pre-intervention), at six weeks and three months after the intervention. Parents were asked to respond to the Chinese version of the parental acceptance-rejection scale, parental stress scale, and self-report scale on parent–child relationship in respect of the transition from kindergarten to primary school.

### Data analysis

To minimize attrition bias, intention-to-treat analysis was done with missing data substituted by the last-observation-carried-forward procedure. The Statistical Package for Social Sciences (SPSS) software, version 18.0 for Windows was used for data analysis. The homogeneity of the experimental and control groups regarding the demographic and baseline data were assessed using inferential statistics (independent *t*-test and chi-square). Mixed between-within subjects ANOVA (split-plot ANOVA) was used to determine which intervention was more effective in enhancing parent–child relationships, helping parents to engage in less harsh parenting practices, and reducing the level of parental stress during the transition from kindergarten to primary school.

## Results

**Figure 1:**
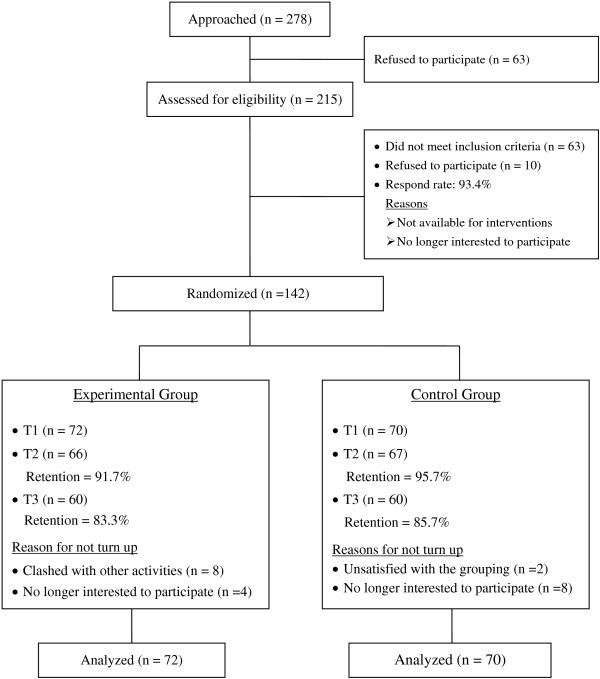
The consolidated standards of reporting trial (CONSORT) flowchart to track participants through randomized controlled trial.

A total of 215 parents living in Tung Chung were approached and assessed for eligibility to participate in the study. Of 215 families, 152 met the inclusion criteria. However, 10 parents were unable to participate because they were not available for the intervention during the scheduled period. The remaining 142 parents were randomly assigned to the experimental and control groups, with 72 parents in the experimental group and 70 parents in the control group. The response rate was 93.4%. The overall retention rates for the experimental and control groups were 83.3% and 85.7%, respectively. Nevertheless, the attendance rates for the intervention sessions were low, with only 48.6 per cent of the experimental group attending all intervention sessions, with 15 participants (42.9%) absent from one, two or three sessions. A Consolidation Standards of Reporting Trials (CONSORT) flowchart is shown in Figure 1. Information on the demographic and baseline characteristics of the experimental and control groups is presented in Table 2. The experimental and control groups were similar with respect to the demographic and baseline characteristics, suggesting a high level of homogeneity of variance between these two groups.

**Table 2:** **Demographic and baseline characteristics of the experimental and control groups (*N *= 142)**

	***n *** **(%)**		***χ***^**2**^	***p***
	**Experimental (n = 72)**	**Control (n = 70)**		
Gender of parent				
Male	8 (11.1)	10 (14.3)	0.10	0.75 ^*ns*^
Female	64 (88.9)	60 (85.7)		
Age range for parents				
20-29	7 (9.7)	8 (11.4)	5.44	0.14 ^*ns*^
30-39	46 (63.9)	34 (48.6)		
40-49	14 (19.5)	25 (35.7)		
50 or above	5 (6.9)	3 (4.3)		
Parents’ education attainment				
No formal education	3 (4.2)	2 (2.9)	0.60	0.96 ^*ns*^
Primary	4 (5.6)	5 (7.1)		
Lower secondary	26 (36.1)	23 (32.9)		
Upper secondary	32 (44.4)	34 (48.5)		
Tertiary	7 (9.7)	6 (8.6)		
	*M* (*SD*)		*t*	*p*
Harsh parenting	35.61 (7.62)	35.44 (7.40)	0.13	0.89 ^*ns*^
Parent–child relationship	8.31 (1.31)	8.31 (1.20)	−0.04	0.96 ^*ns*^
Parental stress	67.54 (6.34)	66.31 (6.23)	1.17	0.25 ^*ns*^

^*ns*^*=* Not significant at *p* > 0.05.

The results of mixed between-within subjects ANOVA on perceived parental aggression, parent–child relationship, and parental stress scores across the three time periods are shown in Table 3. The results indicated that parents in the experimental group engaged in statistically significant less harsh parenting practices and reported better parent–child relationships than parents in the control group. Using the commonly used guidelines proposed by Cohen [38], the eta squared of 0.01, 0.06, and 0.14 are typically interpreted as small, moderate, and large effect sizes respectively. The results suggest moderate-to-large intervention effect sizes. Post-hoc test, using Tukey procedure, confirmed that the differences between groups were statistically significant in both six weeks and three months after intervention. Nevertheless, the results of mixed between-within subjects ANOVA showed that there was no statistically significant difference in parental stress scores between the experimental and control groups.

**Table 3:** **The results of mixed between-within subjects ANOVA on harsh parenting scores, parent–child relationship scores and parental stress scores in parents across the three time periods (*N *= 142)**

	**Harsh parenting**				**Parent–child relationship**				**Parental stress**			
	***F*-value**	***p*-value**	**Eta squared**	**Observed power**	***F*-value**	***p*-value**	**Eta squared**	**Observed power**	***F*-value**	***p*-value**	**Eta squared**	**Observed power**
Time effect	18.13	0.00*	0.27	1.0	6.35	0.00*	0.08	0.89	29.07	0.00*	0.29	1.0
Interaction effect	10.49	0.00*	0.13	0.98	7.51	0.00*	0.10	0.94	3.15	0.04*	0.04	0.65
Intervention effect	10.63	0.00*	0.07	0.89	16.35	0.00*	0.11	0.98	2.29	0.08	0.03	0.40

Notes: *Significant at *p* <0.05.

## Discussion

The overall results provide support for the effectiveness of implementing a parental training programme to enhance parent–child relationship and reduce harsh parenting practices and parental stress in preparing children for the transition to primary school.

### Effect of the parental training programme on harsh parenting practices and parent–child relationship

The results of mixed between-within subjects ANOVA on harsh parenting practices and parent–child relationships indicated that there was a statistically significant main effect for time. That is, there was a change in parents’ harsh parenting practices and the parent–child relationships in both groups across the three different time periods. There was also a statistically significant interaction between time and the intervention, indicating that the changes in parents’ harsh parenting practices and the parent–child relationships over the different time periods differed between the experimental group and the control group. Greater change across different time periods was found in the experimental group than in the control group. In comparison to parents in the control group, parents in the experimental (intervention) group engaged in significantly less harsh parenting practices and reported better parent–child relationships. There are some factors that shed light on these findings. It has been well documented that Hong Kong Chinese parents are influenced by the social-cultural emphasis on obedience, social conformity and academic performance [39]. Consequently, many parents in Hong Kong engage in harsh parenting practices in rearing their children. Although some parents do realise the importance of positive parenting, many of them find it difficult to refrain from engaging in harsh parenting practices. One of the major objectives of the parental training programme was to increase parents’ awareness of risk and reinforce in their minds the consequences of harsh parenting. Role playing, demonstration and re-demonstration not only provided opportunities for parents to act out their roles in front of group members, but also helped them structure an appropriate strategy to refrain from engaging in harsh parenting practices and to maintain positive parenting. Most importantly, parents were able to refine the plan and their skills and enjoy more self-confidence in actualising the plan with their children. This may explain why parents in the experimental group engaged less harsh parenting practices in rearing their children.

Transition creates a time of vulnerability for the parent–child relationship. Previous reports have pointed out that parents who adopt harsh parenting practices and criticism in rearing their children trigger child–parent conflicts and thus hamper the relationship between themselves and their children [6,7]. Therefore, we reasoned that if parents engage in less harsh parenting practices in rearing their children, the parent–child relationships would be enhanced.

### Effect of the parental training programme on parental stress

The results of the mixed between-within subjects ANOVA indicated that there was a statistically significant main effect for time. This finding suggests that there was a reduction in the level of parental stress following the transition to primary 1 (6 weeks and 3 months after the intervention), regardless of the type of intervention given. This is understandable because the parents and their children had already gone through the transition, and some of the problems encountered during the transition had been overcome; thus, the parents’ stress might have decreased. Yet, there was a statistically significant interaction between the time and intervention, which was revealed by the fact that the changes in the levels of parental stress over time were dissimilar between the experimental group and the control group. Parents in the experimental group had lower levels of stress than parents in the control group. Nevertheless, the differences were not statistically significant. The effect size for the intervention was relatively small (0.03), and the result of the power analysis was only 0.40, indicating a high chance of a type II error (60%). Low statistical power resulted in a non-significant difference when, in fact, the intervention might have been effective. The results suggest that the relationship between the particular intervention and parental stress might have been affected by the small sample size. Therefore, it would be interesting to see whether further studies involving larger samples would uncover a significant relationship between the parental training programme and parental stress.

There may be several other reasons for this non-significant finding in terms of the effect of parental training programme on parental stress. One possible explanation lies in the difficulty of determining whether we can even expect parental stress to respond to the parental training programme; the programme did not involve teaching parents any stress management skills. The training programme had a more specific effect in helping parents refrain from harsh parenting practices and enhancing the parent–child relationship. Parental stress as an outcome measure might be less responsive to the intervention.

The baseline parental stress and harsh parenting scores were clinically elevated. One possible explanation is that the stress levels and behaviour of parents might have changed markedly before the transition. Although we did not obtain any information about parents’ stress levels and behaviour at an earlier time, we expected that these outcome measures would have returned to normal with removal of the stressor (i.e., with the children having already transitioned to primary school). However, this study was designed to examine the net differences in mean scores between the experimental and control groups. The complete randomisation should have been able to eliminate such a confounding effect because the extent of decrease in outcome measures in both groups should have been similar. Therefore, the net differences in the stress and the parenting practices of participants in the experimental and control groups should be attributable mainly to the intervention effect.

The overall results provide evidence that the parental training programme was effective in facilitating a smooth transition from kindergarten to primary school. In fact, there was generally positive feedback from parents after their participation in the programme. One parent commented as follows:

‘I did not realise the negative effects of harsh parenting on children before attending the programme. Now, I will use more praise and encouragement instead of harsh parenting practices in rearing my child.’

### Limitations

This study was limited in that all data were collected in a district in Hong Kong, and a small, convenience sample was used, which might limit its generalizability. A multi-district, with larger sample size to examine the effectiveness of the parental training programme to helping their children attain a smooth transition is recommended in future studies. Another limitation of this study was that a simple two-item measure was adopted to assess the parent–child relationships, and the psychometric properties of this measure were not comprehensively examined before use. This limitation may affect the legitimacy of the findings and result in misleading numbers. A validated measure should be used to assess the parent–child relationship in future studies. Additionally, no observation measure was included in the study to assess whether the children moved smoothly into primary school. In Hong Kong, young children attend primary schools based on the geographical location of their homes. The teachers who work in primary schools are normally not the same teachers who work in the kindergartens. Therefore, information provided by teachers from either the kindergartens or primary schools may reflect whether the children better adapt to primary school life after the intervention. It is recommended that researchers consider the possibility of gaining access to both kindergartens and primary schools to obtain more reliable data by introducing observation measures in future studies.

### Practical implications

The findings of this study have important implications for future practice. The results showed that many parents adopted harsh parenting skills in rearing their children during the transition period, which created a time of vulnerability for the parent–child relationship. The most important implications for practice from this study relate to the determination of the effectiveness of the parental training programme to enhance parent–child relationship and reduce harsh parenting practices in the preparation of children for transition to primary school. On the other hand, results showed that a child’s transition from kindergarten to primary school may create considerable stress for the parents. Apart from worrying about their children’s academic achievement and school performance, many parents reported that they had difficulty in accepting the role of teaching and helping their children deal with the emotional frustrations that may emerge during the transition period. Teaching parents some stress management skills in the preparatory parental training programme is therefore a matter of crucial importance. Indeed, problems that arise from a child’s transition to primary school are common around the world. Thus, health educators and healthcare professionals in other parts of the world may incorporate the findings from this study when developing and evaluating an appropriate programme to facilitate a smooth transition for children to primary school life.

## Conclusion

This study has addressed a gap in the literature by providing empirical evidence that the parental training programme is effective in helping parents engage in less harsh parenting practices in rearing their children, and enhancing parent–child relationships towards the transition from kindergarten to primary one. Most importantly, this study highlights the significance of parental involvement in promoting a smooth passage to a pleasurable learning life for children in the primary school.

## Competing interests

The authors declare that they have no competing interests.

## Authors’ contributions

All authors contributed to the study design. WHCL and YWM are responsible for patient recruitment, data collection and data analysis. WHCL is responsible for writing manuscript. THL and SSSC are responsible for reviewing, proofreading and editing the manuscript. All authors contributed to and approved the final manuscript.

## Pre-publication history

The pre-publication history for this paper can be accessed here:

http://www.biomedcentral.com/1471-2458/13/1079/prepub
